# Hippocampal Expression of Connexin36 and Connexin43 during Epileptogenesis in Pilocarpine Model of Epilepsy

**DOI:** 10.18869/acadpub.ibj.21.3.167

**Published:** 2017-05

**Authors:** Sahel Motaghi, Mohammad Sayyah, Vahab Babapour, Reza Mahdian

**Affiliations:** 1 Department of Physiology and Pharmacology, Pasteur Institute of Iran, Tehran, Iran; 2 Department of Basic Sciences, Faculty of Veterinary Medicine, Shahid Bahonar University of Kerman, Kerman, Iran; 3 Department of Physiology, Faculty of Veterinary Medicine, University of Tehran, Tehran, Iran; 4 Department of Molecular Medicine, Biotechnology Research Center, Pasteur Institute of Iran, Tehran, Iran

**Keywords:** Connexin36, Connexin43, Epilepsy, Pilocarpine

## Abstract

**Background:**

Gap junctions (GJs) provide direct intercellular communications that are formed by hexameric protein subunits, called connexin (Cx). The role of Cxs in epileptogenesis has not received sufficient attention. Hippocampus with a critical function in epileptogenesis has a wide network of GJs. We examined the protein expression levels of hippocampal Cx36 (the prominent Cx present between GABAergic interneurons) and Cx43 (the main Cx expressed by astrocytes) during epileptogenesis in the pilocarpine model of epilepsy.

**Methods:**

Male Wistar rats received scopolamine (1 mg/kg, s.c.). Pilocarpine (380 mg/kg, i.p.) was administered 30 min thereafter to induce status epilepticus (SE). SE was stopped 2 h later by diazepam (10 mg/kg, i.p.). Cx36 and Cx43 protein expression was assessed by Western blot analysis in the hippocampus of SE-experienced rats, after injection of diazepam (F0 subgroup), after acquisition of focal seizures (F3 subgroup), and after development of generalized seizures (F5 subgroup). The control subgroups, C0, C3, and C5, were aged-matched rats, which received saline (1 ml/kg, i.p.) instead of pilocarpine. Injection of scopolamine and diazepam, and dissection of hippocampi were carried out at the same time interval as the test subgroups.

**Results:**

SE emerged in 67.1% of pilocarpine-treated animals. Focal and generalized seizures developed 3.8±0.4 and 7.0±0.5 days after SE, respectively. Cx36 protein abundance was not significantly different between test and control groups in the three time points. However, Cx43 protein level showed 40% increase in F3 subgroup (*P*<0.05 compared to C3, *P*<0.01 compared to F0 and F5).

**Conclusion:**

Hippocampal Cx43 is overexpressed in pilocarpine model of epileptogenesis after acquisition of focal seizures.

## INTRODUCTION

Epilepsy is the third most common neurologic disorder after stroke and Alzheimer’s disease[1]. Epilepsy is characterized by spontaneous recurrent seizures. Mesial temporal lobe epilepsy (MTLE) is the most prevalent form of complex focal seizures in which the majority of seizures arise from the mesial temporal lobe structures, especially the hippocampus, amygdala, and parahippocampal gyrus[2]. Epileptogenesis is a process by which different parts of a normal brain are converted to a hyperexcitable, epileptic brain.

Gap junctions (GJs), so-called electrical synapses, are channels that underlie direct electrical communication between adjacent cells and therefore alter neuronal activity over a much shorter period than chemical synapses. GJs are formed by connexins (Cxs), present between neurons and astrocytes, and contribute to the generation, spreading, and maintenance of seizures by synchronization of neural output[3]. The blockade of GJ communication inhibits seizures in different epilepsy models both *in vitro* and *in vivo*[4,5]. Moreover, the expression of Cxs is changed during epileptogenesis[6,7].

One of the main brain regions involved in epileptogenesis is hippocampus, possessing a network of GJ communication between different cell types[8]. The neuro-specific Cx36 mediates electrical coupling between hippocampal inhibitory GABAergic interneurons[9-11]. GJs between inhibitory interneurons are thought to mediate synchronous firing, thereby promoting inhibitory transmission[12]. Propagation of activity within the inhibitory syncytium via GJs will increase inhibitory activity and limit seizure spread[13]. On the other hand, astrocytes are non-neuronal cells that contribute to the control of neuronal function under normal and pathological conditions. The main Cx in the astrocytes is Cx43[14,15]. Astrocytic GJs affect spontaneous epileptiform activity and threshold for eliciting seizure activity as the selective inhibition of Cx43 astrocytic GJs attenuates spontaneous recurrent epileptiform activity in rat organotypic hippocampal slice cultures[4]. Therefore, Cx36 as a neuronal and Cx43 as a non-neuronal Cx seem to be appropriate targets for antiepileptic drugs. It has been shown that changes in Cx36 and Cx43 expression are preceded by epilepsy[8,16]. Nevertheless, there are few and paradoxical reports regarding the role of the two Cxs during epileptogenesis[6-8]. We have previously reported that blocking rat hippocampal Cx36 accelerates epileptogenesis in amygdala kindling[17], as well as hippocampal kindling[18] models of epilepsy.

The pilocarpine model of epilepsy is the most widely-used model to explore molecular mechanisms of epileptogenesis. In this model, epileptogenesis is triggered by status epilepticus (SE), which is induced by pilocarpine. Pilocarpine model replicates several features of MTLE, including similarities in pathology, behavioral abnormalities, and the occurrence of both focal and generalized seizures[19].

There is no report regarding the pattern of Cx36 and Cx43 expression during epileptogenesis in the pilocarpine model of epilepsy, as the most identical model to human MTLE. Therefore, in the present study, we have investigated the changes in the expression of Cx36 and Cx43 in rat hippocampus during epileptogenesis in the pilocarpine model.

## MATERIALS AND METHODS

### Pilocarpine model of epilepsy

Adult male Wistar rats (200-250 g, Pasteur Institute of Iran, Tehran) were used in this study. The animals were kept under 12-h light/dark cycle with free access to food and water. The animal experiments protocol was approved by the Review Board and the Ethics Committee of Pasteur Institute of Iran and conformed to the European Communities Council Directive of November 1986 (86/609/EEC). Pilocarpine (380 mg/kg, i.p., Sigma, Germany) was injected to the animals to induce SE. In order to minimize peripheral cholinergic effects, the rats were pretreated with scopolamine methyl nitrate (1 mg/kg, s.c., Sigma, Germany) 30 min before pilocarpine administration. Diazepam (20 mg/kg, i.p., Sigma, Germany) was administered 2 h after the occurrence of SE to stop it. The animals, in which SE did not lengthen for 2 h, were excluded from the study. The animals showing SE were not able to eat or drink for 2-3 days and received saline i.p. every 12 h. They were then monitored continuously (during day and night) for 2 weeks by using digital cameras (Infra Red CCD Unicorn UN-112S, China) for detection of seizures. Seizures were ranked as score 1: staring with mouth clonus, score 2: automatisms, score 3: unilateral forelimb clonus, score 4: bilateral forelimb clonus, score 5: bilateral forelimb clonus with rearing and falling, and score 6: tonic-clonic seizures[19]. The scores 1-3 were considered as focal seizures, while the scores 4-6 were considered as secondarily generalized seizures.

Animals were divided into two groups as test and control. Each group was composed of three subgroups, each containing six rats. In the test subgroups, pilocarpine was injected to all rats, and left hippocampus of each rat was dissected subsequent to the following events: diazepam injection (F0 subgroup), occurrence of focal seizures (F3 subgroup), and appearance of at least two repetitions of spontaneous generalized seizures (F5 subgroup). The hippocampus was removed not later than 12 h after the occurrence of the focal seizures in F3 subgroup and the generalized seizures in F5 subgroup. The control subgroups were rats with the same age as test subgroups, namely C0, C3, and C5. All rats in control subgroups received saline (1 ml/kg, i.p.) instead of pilocarpine. The injection of scopolamine and diazepam and dissection of left hippocampi were then performed at the same time intervals as the test subgroups. The hippocampi of both test and control groups were immersed in liquid nitrogen and were kept in -80°C. The right hemisphere containing hippocampus was fixed in 10% formalin for histological experiments.

### Immunoblotting

The protein expression of Cx36 and Cx43 in the hippocampus was determined by Western blot technique, which has previously been explained in detail[6]. In brief, the total protein concentration of the homogenized hippocampi was determined by the Bradford assay. Equal amounts of protein from each sample (5 μg per lane for α-tubulin, 10 μg per lane for Cx36, and 25 μg per lane for Cx43) were transferred to a polyvinylidene difluoride membrane (Roche, Germany) by electroblotting. The membrane was blocked in TBST buffer (100 mM Tris base, 150 mM NaCl, and 0.2% w/v Tween 20) and then incubated with the following primary antibodies: mouse monoclonal anti-Cx36 (1:2000 dilution; Zymed, USA), anti-Cx43 (1:100,000 dilution; Upstate, USA), and mouse monoclonal anti-α-tubulin (1:200,000 dilution; Invitrogen, USA). After washing, the membrane was incubated with peroxidase conjugated goat anti-mouse IgG (1:50,000, 1:200,000, and 1:2,000,000 dilutions for Cx36, Cx43, and α-tubulin, respectively; Sigma-Aldrich, Germany). The membrane was then washed with TBST buffer and reacted with electrochemiluminescence advance Western blotting detection reagents (Pharmacia Amersham, UK). Bands were visualized on X-ray film and quantified by densitometry. The relative levels of Cx36 and Cx43 proteins were expressed as ratios (Cx43/α-tubulin×100, Cx36/α-tubulin×100).

### Histopathology experiment

The fixed brains were cut into 2-cm^3^ segments, placed in a tissue processor apparatus (Shanon-Elliott, USA) and processed in the following order: 10% formalin for 6 h, 96% ethanol for 3 h, 100% ethanol for 3 h, xylene for 3 h, and paraffin 63°C for 7 h. The processed segments were molded by paraffin 63°C in metal boxes. The paraffin sections were cut into 5 µm-thick slices by a microtome (Leica instruments, Germany) and stained with hematoxyline and eosin[20]. The cells in CA1, CA3, and dendate gyrus, which had bright and healthy nucleus, were counted manually using a stereoscopic microscope (Olympus, Japan) and a light microscope (Zeiss, Germany). Digital images were taken by a video camera linked to a computer with Motic images software (Motic China Group Co., China). The slices were between -3.24 mm and -5.88mm distance from bregma.

### Statistical analysis

Data were presented as mean±S.E.M. Statistical analysis was carried out using one-way ANOVA with Tukey’s post test. The significance level was considered statistically at *P*<0.05.

## RESULTS

### Temporal lobe epilepsy development

The injection of a single dose of pilocarpine induced SE in 67.1% (34 out of 51) of the animals, and the mortality rate was 47% (16 out of 34). Rats showed SE after a latency of 44.6±2.7 min subsequent to pilocarpine administration. Focal seizures were developed 3.8±0.4 days after SE, while generalized seizures appeared 7.0±0.5 days after SE.

### Immunoblot analysis

**Fig. 1:**
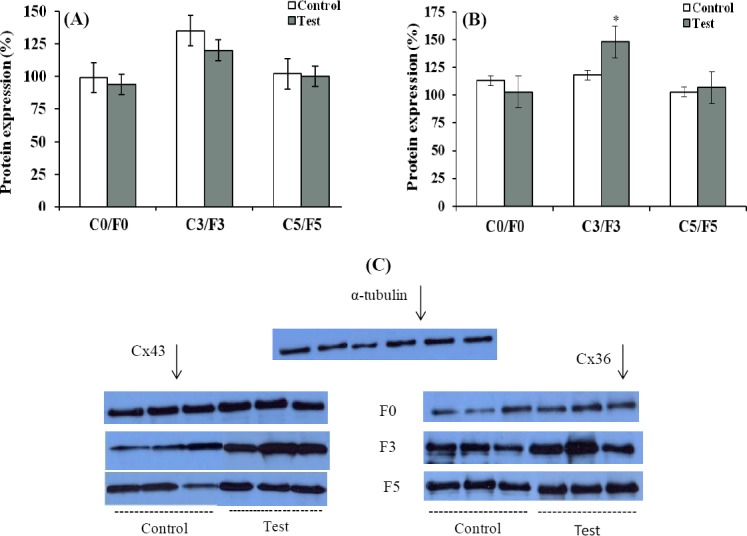
The protein abundance of connexins in the hippocampus of pilocarpine-treated rats. (A) Connexin 36; (B) Connexin 43. F0, after status epilepticus; F3, after development of focal seizures; F5, after acquisition of generalized seizures. C0, C3, and C5 are control time points matched to F0, F3, and F5, respectively. Cx36 and Cx43 protein levels were normalized to the level of α-tubulin protein. Data are expressed as means±S.E.M (n=6). Panel C shows representative immunoblots of Cx43 and Cx36 in the groups. ^*^P<0.01 compared to F0 and F5 test subgroups.

Cx36 protein abundance in C3 subgroup was higher than the levels in C0 and C5 subgroups (Fig. 1A). However, the difference between the subgroups was not statistically significant (*P*>0.05). There was not any significant difference (*P*>0.05) between the test subgroups (F0, F3, and F5) in expression of Cx36 protein (Fig. 1A and 1C). Cx43 protein in the F3 subgroup showed a significant increase compared to respective control group C3 (*P*<0.05, Fig. 1B and 1C). Moreover, Cx43 protein level at this subgroup was almost 40% higher than that in F0 subgroup (*P*<0.01), and in F5 subgroup, it was at the same level as F0.

### Histopathology

**Fig. 2:**
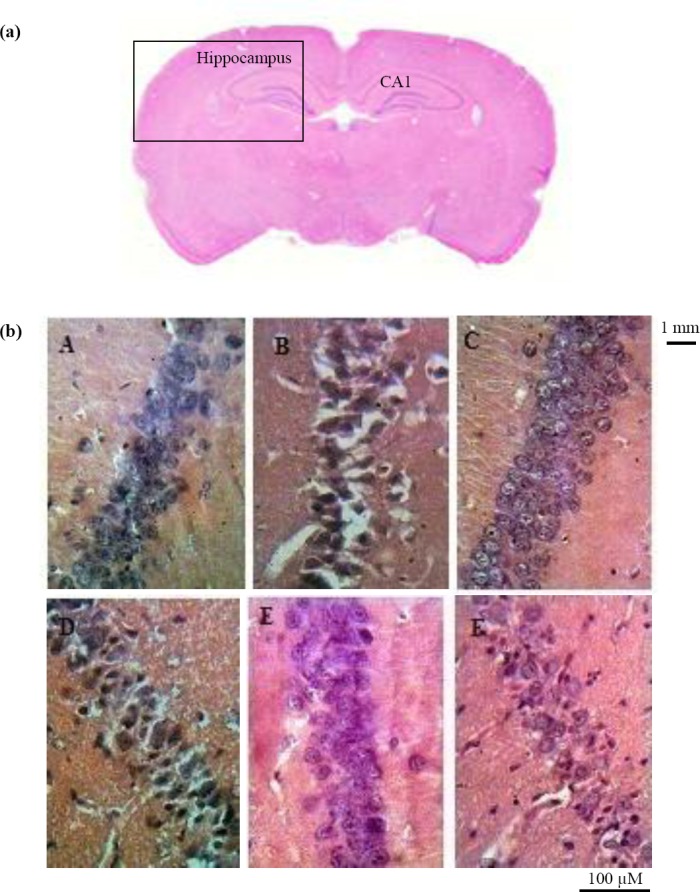
Neurodegenerative changes in the hippocampus of pilocarpine-treated rats. (a) coronal section of rat brain in control group; (b) higher magnification of the section seen in part a; Neurodegeneration in CA1 region of hippocampus (20 × objective lens) in control (A, C0; C, C3; E, C5) and pilocarpine-treated rats (B, F0; D, F3; F, F5). Significant neural loss is observed in CA1 area of the hippocampus in F0 group versus control subgroups. F0, after status epilepticus; F3, after development of focal seizures; F5, after acquisition of generalized seizures.

Highly neurodegenerative changes were observed in the hippocampus of pilocarpine-treated rats. The changes were visible in all regions, particularly in CA1 subfield (Fig. 2). The number of cells was decreased significantly in CA1 region in F0 group compared to respected control group (383.3±149.0 versus 2604.5±49.5, *P*<0.01). Morphological changes in neurons were observed as perikaryon shrinking and chromatin condensation, which are typical of neurodegenerative process. The cell loss was also considerable in the CA3 and dendate gyrus regions.

## DISCUSSION

The present study indicates some neurodegenerative changes in the hippocampus of pilocarpine-treated rats. The changes were visible in all hippocampal regions, particularly in CA1 area, which is in line with the previous study that indicated immediate neuronal damage in the CA1 field of hippocampus in pilocarpine model of epilepsy[19]. This significant hippocampal neurodegeneration has been attributed to microcirculation defects induced by pilocarpine, which results in tissue hypoxia followed by necrosis[19].

In the CA1 subfield of hippocampus, paravalbumin-positive GABAergic interneurons form a vast dendrodendritic network, which is responsible for synchronized oscillations in hippocampus and thereby promote inhibitory transmission[12]. Electro-physiological[12] and morphological[9] evidence indicate the presence of electrical coupling between GABAergic interneurons in this region, which is mediated by Cx36. Nonetheless, neuronal and non-neuronal cells other than GABAergic interneurons might also express Cx36 in the hippocampus. GJ communication can be regulated at several levels such as changes in Cx transcription, translation, stability, post-translational processing, and channel gating. However, post-translational processes are thought to be a major factor in regulating Cxs levels and functional coupling[21]. In the present study, Cx36 expression did not undergo any significant change at post-translation level during entire period of epileptogenesis. Therefore, it is unlikely that Cx36 GJ communication is distorted in the pilocarpine model of epilepsy. In order to confirm this proposal, further complementary studies are required regarding Cx36 channel gating during epileptogenesis and the effect of specific Cx36 blockers on the rate of epileptogenic process in the pilocarpine model. On the other hand, we have previously reported that Cx36 expression is increased in the hippocampus during epileptogenesis in the electrical kindling model of epilepsy at both mRNA and protein levels[6]. The discrepancy in the results can be due to the different animal models used in these studies. In 1980s, electrical kindling was considered as a well-established model of TLE. However, with the increased knowledge on animal models, it is not currently considered as a model of TLE since TLE is characterized by recurrent, difficult-to-treat seizures, and hippocampal pathology, which are not mimicked by kindling. However, pilocarpine and kainite models of epilepsy greatly resemble TLE in human[19]. In contrast to electrical kindling, systemically administered chemoconvulsants, such as pilocarpine, cause hippocampal and extra-hippocampal damages[19]. Nonetheless, GABAergic inhibition remains functional and intact in the CA1 pyramidal cell layer of pilocarpine-treated rats[22]. Thus, it seems that neurodegenerative changes in the hippocampus induced by pilocarpine do not concern Cx36 protein abundance.

We found that hippocampal Cx43 protein expression is increased after the acquisition of focal seizures, and then it returns to control level when the animals become fully epileptic and develop generalized seizures. In agreement with this finding, Takahashi *et al*.[23] showed that Cx43 protein was significantly overexpressed early in the process of epileptogenesis in the kainic acid model of TLE. Our observation has also been supported by the previous studies that indicated epileptic activity is accompanied by increase in Cx43 protein abundance in both human[16] and animals[24,25]. In a similar study to our work, Wu *et al*.[25] have recently reported that the expression of Cx43 in mouse hippocampus is increased at both mRNA and protein levels in the pilocarpine model of epilepsy at 1 week and 2 months but not 4 days after the induction of SE. In contrast, we found a significant increase in the hippocampal expression of Cx43, four days after SE (the time point in which the rats developed focal seizures), and no changes were seen in the hippocampal expression of Cx43 at one week after SE (the time point in which the rats developed generalized seizures). The reason of contradictory results obtained in these two studies might be that we stopped pilocarpine-induced SE after two hours by injection of diazepam to minimize the level of neuronal death. However, Wu *et al*.[25] did not stop SE. Moreover, they used mice, while we evaluated Cx expression in rats. In another animal model of epilepsy, electrical kindling, we have previously shown that Cx43 expression is not changed in the hippocampus[6]. Elisevich *et al*.[7] also reported no change in Cx43 expression in amygdala of rats during development of amygdala kindling. These findings are also in contrast to the results of the present study in which the pilocarpine model of epilepsy is used. The opposite finding can be attributed to fundamental differences between these two discrete models of epilepsy i.e., pilocarpine and electrical kindling.

It remains to be explored whether overexpression of Cx43 facilitates epileptogenesis, or it is rather a compensatory response to modify the process. Astrocytic and microglial activation, which is elicited by pilocarpine-induced SE, underlies epileptogenic mechanisms. Some researchers reported that the inhibition of GJ coupling between glial cells by Cx43-mimetic peptides suppresses spontaneous seizures[4]. However, studies in double-knockout mice for Cx30 and Cx43 (Cx30^-/-^Cx43^-/-^) suggest a major role for astrocytic networks in glutamate clearance, potassium buffering, and extracellular space-volume regulation during basal synaptic activity. In Cx43 and Cx30 knockout mice, due to decreased astroglial glutamate and potassium clearance, the threshold of epileptiform discharge is reduced[26], and hippocampal synaptic transmission and neuronal excitability are increased[15]. In addition, it has been reported that Cx32-Cx43 knockout mice develop seizures[27]. Therefore, upregulation of Cx43-containing GJs in our study seems to be an adaptive response to neuronal hyperexcitability in order to reestablish extracellular homeostasis and thereby regulate neuronal activity. These possibilities can be further addressed by studying the rate of epileptogenesis in conditional Cx-deficient mice and by using new highly specific GJ blockers.

In summary, we showed that hippocampal GJs, composed of Cx43, are up-regulated during the acquisition of focal seizures in the pilocarpine model of TLE. The upregulation seems to be an adaptive response to excitotoxic stimuli induced by SE, to redistribute the imbalance in extracellular ions and neurotransmitters throughout the astrocytic network. The balance of ions and neurotransmitters between inside and outside of the neurons in turn can modulate neuronal excitability. However, double staining immunohistochemistry should be carried out to verify the cellular (neuronal or astrocytic) origin of the overexpressed hippocampal Cx43 observed in our study. Furthermore, *in vivo* application of highly specific Cx43 blockers during epileptogenic process might further clarify the significance of Cx43-containing GJs in development of epilepsy.
